# The Impaired Response Inhibition and Salience Attribution Model of Drug Addiction: Recent Neuroimaging Evidence and Future Directions

**DOI:** 10.1146/annurev-psych-040725-025923

**Published:** 2025-10-01

**Authors:** Ahmet O. Ceceli, Yuefeng Huang, Greg Kronberg, Natalie McClain, Sarah G. King, Eduardo R. Butelman, Nelly Alia-Klein, Rita Z. Goldstein

**Affiliations:** 1 Department of Psychiatry, Icahn School of Medicine at Mount Sinai, New York, NY, USA; 2 Department of Neuroscience, Icahn School of Medicine at Mount Sinai, New York, NY, USA

**Keywords:** addiction, substance use disorders, prefrontal cortex, salience attribution, inhibitory control, craving

## Abstract

Originally postulated in 2001, the impaired response inhibition and salience attribution (iRISA) model of addiction highlights the prefrontal cortex (especially the orbitofrontal, dorsolateral, anterior cingulate, and inferior frontal regions) as central to drug addiction symptomatology. Accordingly, drug cues assume a heightened salience and value that overpower alternative reinforcers, with a concomitant decrease in inhibitory control, especially in a drug-related context. These processes may manifest in metacognitive impairments (e.g., self-awareness of choice), obstructing insight into illness, as a function of recency of drug use. In this review, we update the neurobehavioral evidence for iRISA two decades later, emphasizing the robust measurement of the iRISA interaction (between a drug-related cue/context and a cognitive-behavioral function), and highlight relevant individual differences (e.g., drug use severity, craving). Crucially, we describe data suggesting functional recovery (with abstinence, treatment, and other emerging modalities) and the need for identifying valid outcome biomarkers. We end by highlighting recent developments in artificial intelligence (e.g., natural language processing applied to spontaneous speech) and computational modeling, and call for enhanced ecological validity to facilitate dynamic and clinically meaningful neural explorations in drug addiction.

## 1. INTRODUCTION

### 1.1. The Subcortex-Centered View of Drug Addiction

Early neurobiological studies in addiction were bolstered in the 1950s by the observation that electrical activation of several subcortical structures (e.g., in the septum, as well as origins and targets of fibers in the medial forebrain bundle) could result in reward-like effects in rodents (Gardner 2011, Olds 1962, Olds & Milner 1954). In the 1960s and thereafter, using mu opioid receptor (MOR) agonists (e.g., heroin, morphine), preclinical studies elucidated pharmacological and operant self-administration mechanisms of drug-induced reward, as well as the basis of dependence and withdrawal (Deneau & Seevers 1964, Deneau et al. 1969). Further advances implicated brain stem sites (e.g., locus coeruleus and its projections) in these addiction-related processes, which serve as important motivators of drug taking in the advanced stages of the disease (Duman et al. 1988). Together, a considerable amount of progress followed the finding that drugs of abuse (e.g., MOR agonists, alcohol, cocaine and other stimulants) cause acute elevations in dopamine dialysates in terminal areas of nigrostriatal and mesolimbic systems (e.g., dorsal and ventral striatal areas such as caudate, putamen, and nucleus accumbens) (Di Chiara & Imperato 1988, Hurd et al. 1990, Pettit et al. 1984, Robbins & Everitt 2002, Unterwald et al. 1992). An initial distinction was made between these acute rewarding effects of drugs versus consequences of chronic drug exposure (associated with increased versus reduced dopamine neurotransmission, respectively). Specifically, the acute rewarding effects and function in the ventral striatum were examined as a model for earlier stages in addiction trajectory. By contrast, more advanced stages in the addiction trajectory implicated dorsal striatal areas, underlying compulsive-like drug-taking behaviors (Fagergren et al. 2003). Translationally, clinical positron emission tomography neuroimaging studies from the 1990s onward used MOR or dopaminergic radiotracers, detecting significant differences in these neurotransmitter functions when people with drug addiction were compared to healthy controls (HCs) (Volkow et al. 1997, Zubieta et al. 1996).

### 1.2. The Emergence of a Prefrontal Cortex Account and the iRISA Model of Drug Addiction

Studies from the 1990s onward focused on the chronically relapsing nature of addiction, exploring the mechanisms that remained dysregulated even after long-term abstinence or drug-free periods. For example, rodent models of the incubation of craving (an increase in drug seeking as a function of withdrawal/abstinence duration) implicated several subcortical areas, including the amygdala and mesolimbic system, but also mesocortical target areas, including in the prefrontal cortex (PFC) (Shaham et al. 2003). Overall, current models of addiction posit an interactive system of several subcortical and cortical areas that are affected at specific stages in the addiction cycle (e.g., initial drug taking, escalation, abstinence/withdrawal, and relapse) (Everitt et al. 2008, Koob & Volkow 2016).

Late in the 1990s, functional neuroimaging work led by Volkow and Fowler highlighted the role of the PFC in human drug addiction. This focus derived from glucose metabolism studies that showed decreases in PFC function in people with drug addiction (e.g., to cocaine, methamphetamine, heroin, alcohol), interpreted to underlie the chronically relapsing nature characterizing this disorder (for a review, see Volkow et al. 2009). Transient increases in glucose metabolism in the orbitofrontal cortex (OFC) in early cocaine abstinence (Volkow et al. 1991), mirroring findings in obsessive compulsive disorder (Swedo et al. 1989), highlighted the potential role of this region in the compromised inhibitory control underlying persistent craving and relapse in addiction. Linking these PFC deficits to dopamine receptor availability suggested an underlying role for striatal neurotransmission in higher-order executive functions in addiction (Volkow et al. 1993, 2001). At the same time, work based on nonhuman primate and rodent behavior emphasized the role of the PFC in core neuropsychological substrates underlying drug addiction (Everitt & Robbins 2005, Jentsch & Taylor 1999). In general, these earlier studies expanded the view of addiction beyond the reward and habit subcortical regions that were (and still are) commonly the focus of preclinical studies and rodent models.

**Figure 1:**
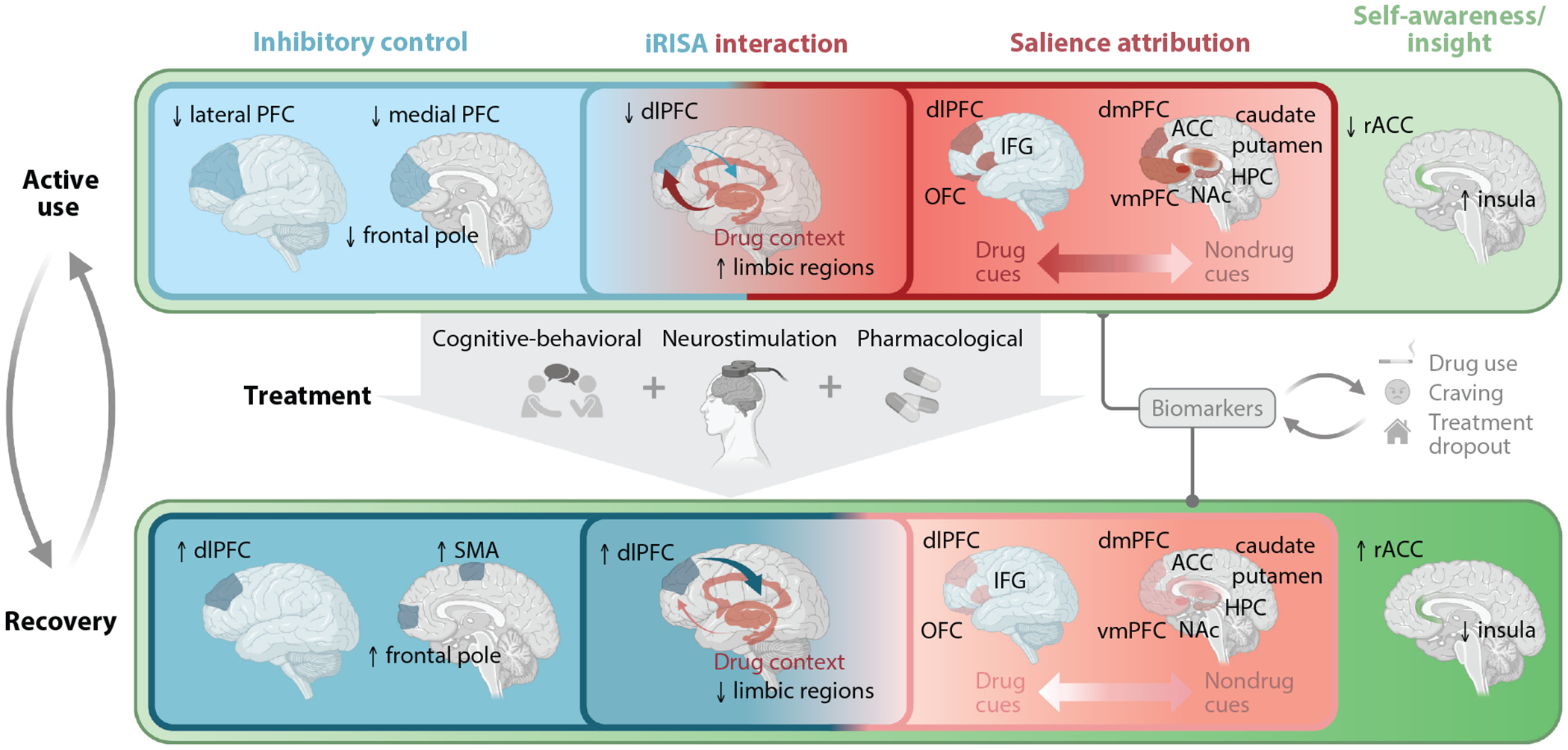
The neural and behavioral substrates of the iRISA model of drug addiction during active use and recovery. The active use phase (*top row*) of the drug addiction cycle is characterized by impaired inhibitory control (and other higher-order executive functions) largely accompanying lower executive PFC engagement (“cold” functions depicted in a *blue gradient*; a paler hue designates more impairment), excessive drug versus nondrug salience attribution as evidenced by the limbic and cortical reactivity in a drug context (“hot” functions depicted in a *red gradient*; a bolder hue designates more drug bias), and the interaction of these core functions as accompanied by limbic drug cue reactivity that interferes with PFC inhibitory control. Enveloping these neurobehavioral aberrances are impairments in self-awareness/insight into illness (*light green*) that exacerbate the cyclical/chronically relapsing nature of drug addiction (depicted with a *bidirectional arrow* between active use and recovery on the *left*). We emphasize different interventions (separately and in combination; *middle row*) en route to recovery (e.g., drug use reduction or cessation). (*Bottom row*) With treatment, inhibitory control functions improve under nondrug and drug contexts (*darker blue*) as the salience attributed to drug cues diminishes (*lighter pink*) in comparison to alternative rewards (*darker pink*). Self-awareness/insight into illness is enhanced (*darker green*), further facilitating recovery. Biomarkers can be identified through this lens (e.g., neural cue reactivity and its change as a function of treatment), allowing for the prediction of clinical outcomes (*middle right*). Abbreviations: ACC, anterior cingulate cortex; dlPFC, dorsolateral prefrontal cortex; dmPFC, dorsomedial prefrontal cortex; HPC, hippocampus; IFG, inferior frontal gyrus; iRISA, impaired response inhibition and salience attribution; NAc, nucleus accumbens; OFC, orbitofrontal cortex; PFC, prefrontal cortex; rACC, rostral anterior cingulate cortex; SMA, supplementary motor area; vmPFC, ventromedial PFC. Figure adapted from images created in BioRender; McClain N. 2026. https://BioRender.com/zpyasxw.

Bridging between the nonhuman animal studies and those of the human brain, in the impaired response inhibition and salience attribution (iRISA) model we emphasized the PFC in these neuropsychological functions as underlying the core clinical symptoms of drug addiction, including craving. Specifically, the iRISA model featured the OFC’s roles in value processing of salient stimuli, and adaptively shifting behavior after environmental contingencies have changed, as underlying the compromised ability to stop or reduce drug use in addiction. Other PFC brain regions were also highlighted, encompassing the anterior cingulate cortex (ACC) in salience attribution and awareness of choice, the ventromedial PFC (vmPFC) in subjective valuation of drug and nondrug reinforcers, and the inferior frontal gyrus (IFG)/ventrolateral PFC and dorsolateral PFC (dlPFC) in inhibitory control. In addition to emphasizing the importance of drug-related cues/contexts as crucial modulators of cognitive function, across all these functions we called for experimental designs that would allow the direct comparison to salient nondrug cues (and not only to the commonly employed neutral cues). Specifically, these designs would aim to test a core tenet of the iRISA model: a seesaw relationship between drug and nondrug cues, where the salience of drug cues dominates over other arousing and valuable reinforcers. This imbalance was postulated to be associated with a marked reduction in the ability to inhibit or control behavior, along with meaningful impairments in other cognitive functions. The cognitive-behavioral deficit (or change) was expected to be pronounced in a drug-related context (i.e., drug cue reactivity) as associated with clinical symptoms (e.g., drug use and craving) (Figure 1). Since the first review that introduced iRISA in 2001 (cited >3,500 times for a cumulative total of >7,000 citations, inclusive of the 2011 and 2018 reviews as per Google Scholar as of the time of writing), hundreds of neuroimaging studies compared people with addiction to HC participants, exploring numerous higher-order cognitive-behavioral and emotional functions encompassing the ones mentioned above.

Here, we review the neuroimaging studies (published from 2018 onward) highlighting PFC deficits across relevant cognitive-emotional functions in people with addiction [and we refer readers to recent reviews and meta-analyses for accompanying structural abnormalities and their recovery (Hill-Bowen et al. 2022, Pando-Naude et al. 2021, Parvaz et al. 2022)]. Then, we focus on studies that approximate the iRISA interaction effect (by directly comparing drug cues to other salient cues during the performance of a neuropsychological task). We further examine the approaches that can ameliorate iRISA, encompassing abstinence, cognitive-behavioral, pharmacological, and direct brain stimulation interventions. We conclude by highlighting novel paradigms that are ecologically valid, naturalistic, scalable, and amenable for artificial intelligence analyses. We argue that these paradigms can optimally serve to identify valid PFC biomarkers and predictors of clinical outcomes while also advancing the fundamental and translational neuroscience study of iRISA in drug addiction.

## 2. CUE REACTIVITY

Drug-biased salience attribution in individuals with substance use disorders (SUDs) presents as cortico-striatal hyperactivations in response to drug cues and hypoactivations in response to nondrug reinforcers (e.g., food), evident across reward, salience, habit, executive, memory, and self-directed networks [see our previous reviews (Ceceli et al. 2022a; Goldstein & Volkow 2002, 2011; Zilverstand et al. 2018)]. With neuroimaging, this impairment is accessible via the cue reactivity task (Ekhtiari et al. 2022; Huang et al. 2018b, 2024), whereby drug-related stimuli (e.g., pictures, videos, audio, paraphernalia, and tastes) are compared mostly to neutral stimuli (and sometimes to other salient reinforcers) across people with nicotine, alcohol, cannabis, cocaine or other stimulant (e.g., methamphetamine), and opioid use disorder as further compared to HCs.

Since our last systematic review (Zilverstand et al. 2018), widespread drug cue–related hyperactivations in cortico-striatal regions (e.g., striatum and medial PFC) were reported in individuals with nicotine (Allenby et al. 2020, Haugg et al. 2022, Janes et al. 2020), alcohol (Huang et al. 2018b, Tan et al. 2023), cannabis (Kuhns et al. 2021), stimulant (Grodin et al. 2019, Huang et al. 2018a, MacNiven et al. 2018), and opioid (heroin and prescription opioid) (Ekhtiari et al. 2021, Shi et al. 2021) use disorders as largely associated with (and potentially exacerbated by) drug craving (for meta-analyses, see Lin et al. 2020, Pollard et al. 2023, Zeng et al. 2021). While these findings suggest a common salience impairment pattern across substances, a recent meta-analysis indicated substance-specific drug cue reactivity patterns, with differences between heroin use disorder (HUD)—associated with more widespread drug cue activations encompassing the ventral striatum, temporal gyrus, thalamus, and other regions—and cocaine use disorder (CUD)—associated with more dlPFC activity (Dejoie et al. 2024). Intriguingly, these functional patterns align with our structural findings on substance-specific frontostriatal gray matter volume differences between these substance classes, whereby in HUD we observed smaller nucleus accumbens/putamen and vmPFC gray matter volumes, while in CUD the IFG was smaller (Ceceli et al. 2023a).

In our last review we also noted a paucity of studies in people with HUD (Zilverstand et al. 2018). This gap has been slowly decreasing, revealing the expected drug cue–related hyperactivations. For example, this pattern was observed in the OFC, dlPFC, and insula [even when compared to salient nondrug reinforcers (sexual cues)], along with drug cue–induced negative connectivity between the dlPFC and the OFC, putamen, and thalamus, in abstinent individuals with HUD (Liu et al. 2021). In abstinent inpatients with HUD, we similarly reported the expected cortico-striatal drug-related (versus neutral or food picture cues) hyperactivations (in the OFC, vmPFC, IFG, and nucleus accumbens) that were correlated (for the vmPFC) with drug craving. Moreover, hyperactivations in the OFC, anterior PFC/IFG, and rostral ACC were observed during drug reappraisal (a top-down emotion regulation technique to reduce drug cue reactivity), where dlPFC activity was correlated with a lower cue-induced drug craving and a higher methadone dosage (Huang et al. 2024). Yet drug reappraisal came at the expense of food savoring (another emotion regulation technique aimed at increased processing of alternative hedonic reinforcers), itself a function of treatment length (i.e., rostral ACC savoring activity was correlated with longer treatment duration). Using a naturalistic stimulus in these same patients (the drug-themed movie *Trainspotting*), we again reported a widespread drug bias encompassing the OFC, vmPFC, IFG, dlPFC, and insula, now measured as a temporally synchronized group signal driven by the drug-related movie scenes (Kronberg et al. 2025). Patterns of the emotion regulation strategies remain to be similarly studied and then implemented as interventions in a personalized and time-sensitive manner.

Notably, very few studies addressed sex differences, partly reflecting the underrepresentation of women in clinical neuroimaging studies in addiction (Zilverstand et al. 2018). Since then, several studies have investigated sex differences in SUDs, particularly in alcohol use disorder (AUD). In these studies, men exhibited more drug cue–related striatal (putamen and caudate) hyperactivations, whereas women exhibited more drug cue–related cortico-limbic (dlPFC, vmPFC, and insula) hypoactivations, as associated with craving and future drug use in the women (Radoman et al. 2024, Smith et al. 2023). Opposite results were also reported encompassing more drug cue–related cortico-limbic hyperactivations in the women than men (Claus et al. 2022). In our recent study, we similarly found that women with HUD or CUD exhibited hyper–drug cue reactivity in the medial PFC, while men with HUD had higher dlPFC/frontal eye field drug reappraisal activity that correlated with lower cue-induced drug craving (Huang et al. 2025). In CUD, sex differences were also observed in the association between craving and drug cue–related connectivity between the vmPFC and periaqueductal gray area, with a positive correlation in men and a negative correlation in women (Zhang et al. 2020). Overall, the variability in results between these studies could be related to task variations (e.g., picture versus imagery scripts, with versus without stress cues or emotion regulation components) as well as substance class differences. Taken together, and considering the epidemic-level overdose mortality rates (Butelman et al. 2023) and nonmedication opioid use in outpatients (Butelman et al. 2025), where vulnerability patterns were higher in men versus women, the continued precise mapping of the impact of sex differences on addiction neurobiology and phenomenology is needed.

Recent studies have also explored hormonal influences within these results. A study in naturally cycling women with nicotine dependence showed that smoking cue–induced ventral striatal hyperactivations were greatest during the late follicular phase (high estradiol) followed by the mid–luteal phase (high estradiol and progesterone) as compared to the early follicular phase (low estradiol and progesterone) (Franklin et al. 2022). Similarly, in women with HUD or CUD, we recently demonstrated higher PFC drug cue reactivity in the follicular phase as associated with higher craving and estradiol levels; higher PFC drug reappraisal activity in the luteal phase was associated with a higher progesterone/estradiol ratio (Huang et al. 2025). Collectively, these studies suggest that estradiol may constitute a vulnerability factor (enhancing drug cue reactivity), while progesterone may serve as a protective factor (enhancing its regulation) in women with addiction. Further efforts that target similar hormonal mechanisms are needed to develop individualized treatments for women with addiction.

Other individual differences have also been examined recently in relation to impaired salience attribution. For example, individuals with CUD who had a history of withdrawal exhibited greater drug cue reactivity (in the OFC, ACC, IFG, dorsomedial PFC, and caudate) than those without such history (Denomme & Shane 2020). Similar associations between cortico-striatal drug cue reactivity and withdrawal symptoms or drug use severity were reported in HUD (Shi et al. 2021). These findings suggest that drug use history and factors beyond craving and sex differences/hormonal effects may influence drug-biased salience attribution in drug addiction.

## 3. INHIBITORY CONTROL (AND OTHER HIGHER-ORDER EXECUTIVE FUNCTIONS)

Inhibitory control, the ability to suppress unwanted or inappropriate behaviors, has been studied in people with SUDs using various experimental tasks. These include the stop-signal task, which is a hallmark measure that estimates the stopping of an initiated response (Verbruggen & Logan 2008), the go/no-go task, which measures response selection (Goghari & MacDonald 2009), and the flanker and Stroop tasks, which index attentional interference (for details on each task, see Ceceli et al. 2022a).

Since our previous reviews, research has further supported the neurobehavioral inhibitory control alterations in drug addiction across substances and as modulated by drug use history and other relevant individual differences. For example, in individuals with CUD, more years of cocaine use, and fewer days since recent use, were associated with worse stopping latency (i.e., longer stop-signal response time, a hallmark measure of inhibitory control) on the stop-signal task (Li et al. 2023); fewer days since last use were also associated with lower inhibitory control PFC activity (Nestor et al. 2019). Extending these patterns to opioid use disorder, we recently revealed worse target detection sensitivity (i.e., the proportion of hits over false alarms) and lower inhibitory control network activity (anterior PFC and dlPFC) during this task in inpatients with HUD; worse stopping performance, fewer days since last use, and higher severity of dependence were associated with more pronounced impairments in these brain regions (Ceceli et al. 2023b). Individuals with AUD also have inhibitory control network hypoactivations during the stop-signal task, this time as a function of age (Swartz et al. 2021), suggesting that these characteristic inhibitory control deficits may become more pronounced with aging [itself suggested to be accelerated in SUDs (Bachi et al. 2017)] ( Jentsch & Pennington 2014). Another developmental factor that may inform inhibitory control alterations is trauma exposure. In veterans with AUD, the dorsal ACC and dlPFC go/no-go task activity positively correlated with trauma distress and negative affect, respectively (Tenekedjieva et al. 2024). Because this study did not report behavioral performance effects, this inhibitory control cortical overreliance may have been needed to normalize task performance as a function of the level of distress. Importantly, these inhibitory control behaviors may be predictive of prospective drug use as reported in people with tobacco use disorder, cannabis use disorder, or AUD, where shorter response times in a daily administered Stroop task (and higher fractional anisotropy, a white matter integrity measure) were associated with a lower probability of next-day substance use (Chirokoff et al. 2024).

Recent research has highlighted differential functional connectivity and network dynamics as underlying inhibitory control deficits in SUDs. For instance, during the stop-signal task, individuals with CUD exhibited lower temporal flexibility (i.e., the frequency of communication with other networks) in frontoparietal task networks and higher spatiotemporal diversity [i.e., uniformity in communication between networks, especially in subcortical and ventral attention networks (Zhang et al. 2018)]. Individuals with CUD also exhibited widespread deficiencies in intrinsic connectivity distribution (reflecting voxel-wise connectivity profiles unconstrained by seed regions of interest) throughout the brain, and especially in the PFC, including the dlPFC, IFG, and OFC during the color-word Stroop task (Zakiniaeiz et al. 2023). Importantly, dynamic functional connectivity may be a particularly sensitive estimate of inhibitory control brain alterations in drug addiction. Here, the dynamic functional connectivity estimate during a stop-signal task classified CUD versus HC groups with 90% accuracy using fewer independent component pairs (i.e., connectivity strength between pairs of networks including cognitive control, sensorimotor, salience, visual, amygdala, or default mode) when compared to the static connectivity method, which yielded 81% classification accuracy with more pairs of components (Sakoglu et al. 2019). Similar functional connectivity abnormalities are also evident at rest: Compared to HCs, individuals with CUD showed abnormalities in local and global efficiency [i.e., the capacity for information transfer between regions (Latora & Marchiori 2001)], as evident in lower executive, semantic, basal ganglia, and salience networks, and higher default mode and visual network efficiency (Zilverstand et al. 2023). Importantly, these functional connectivity abnormalities were associated with worse inhibitory control (assessed with the stop-signal task outside the imaging environment), higher craving, and recency of cocaine use (Zilverstand et al. 2023).

In addition to inhibitory control, investigating the neural basis of decision-making (and related) deficits has provided insights into the general cognitive impairments that exacerbate drug-biased behaviors and hinder recovery in SUDs (Verdejo-García et al. 2006). Reduced coherence and higher intensity in the anterior salience network during resting-state fMRI, driven by the dorsal ACC (which has a role in governing the switch from automatic to controlled processing), were associated with delays in decision-making on the Cambridge Gambling Task in people with AUD (Canessa et al. 2021). Taking into account ACC (and OFC) functions, contributing factors may be blunted error correction rates, reflecting disrupted negative feedback processing (Li et al. 2020) and deficits in learning from negative outcomes (Duehlmeyer & Hester 2019) as seen in smokers. Impairments in learning from punishments (i.e., reduced sensitivity to losses) have also been documented in stimulant addiction (Ahn et al. 2014). A potential underlying culprit may be a difficulty in updating internal states during learning, as suggested by intact cognitive state transition activity in the salience network in HCs that was absent in smokers (Li et al. 2020).

## 4. THE iRISA INTERACTION: COGNITIVE FUNCTION UNDER DRUG CUE REACTIVITY

### 4.1. Inhibitory Control

To date, a majority of the studies that probe iRISA in drug addiction have used classic cognitive neuroscience tasks that isolate inhibitory control and cue reactivity to estimate their respective neurobiological underpinnings. While their study in silo has yielded impactful findings in the field, the interaction of self-control (and other higher-order executive functions) with cue-reactive states remains to be fully explored. With this nontrivial extension, we emphasize the importance of studying the need to override a drug-seeking response under a salient drug-associated context, which is of central relevance to the challenges faced by people with SUDs in the real world (see Figure 1 for the centrality of this interaction in our model).

The iRISA interaction may be accessible with modifications to classic inhibitory control tasks whereby cue-reactive states can be readily integrated. For instance, the trial-by-trial modulation of the color-word Stroop task (i.e., the drug Stroop task, whereby the color-word stimuli are replaced by drug and other words) revealed worse performance and lower dlPFC activity in response to drug (versus neutral) cues in people with AUD, as associated with more years of alcohol use (Müller-Oehring et al. 2019). This drug Stroop effect also showed dynamic changes throughout recovery (DeVito et al. 2018). For example, longer periods of continuous abstinence as a function of treatment in individuals with CUD correlated with greater reductions in the behavioral drug Stroop effect as associated with greater engagement with computer-based cognitive-behavioral therapy (but not with standard treatment) (DeVito et al. 2018). In a similarly modified go/no-go task, inhibitory control during drug cue trials was worse when compared to blank no-go performance, as associated with IFG hypoactivations in individuals with methamphetamine use disorder (Dakhili et al. 2022). In a stop-signal task where drug and nondrug words served as task cues (replacing the usual neutral cues), we revealed lower dlPFC activity during drug compared to food (a nondrug salient cue matched to the drug cues on arousal) trials in people with CUD as compared to HCs (Ceceli et al. 2022b). Overall, these drug cue–induced inhibitory control hypoactivations are indicative of interference by salient drug cues with cortical control during inhibitory performance.

Contrasting patterns are also evident. For example, in AUD, the dlPFC and ACC were hyperactivated to alcohol compared to neutral cues in a go/no-go task, as correlated with increased craving (Stein et al. 2021) with similar rostral ACC hyperactivations on a drug Stroop task (Murray et al. 2022); behavioral performance was variable between these studies (no effect in the former and a drug effect on reaction time in the latter). In another modified stop-signal task, and as compared to HCs, people with CUD had higher OFC and IFG (and subthalamic nucleus) activity during drug compared to neutral cue inhibition despite comparable task performance (Terenzi et al. 2024). Such drug cue–related hyperactivation was also evident in connectivity measures, with higher drug Stroop–related ACC-hippocampus effective connectivity in HUD, increasing as a function of reaction time (Ma et al. 2019), alluding to the activation of hippocampal memories that require cortical control during task performance. Overall, the hyperactivations during inhibitory control under drug cue reactivity may reflect increased effort needed to match the cognitive demand induced by the salient task cues. However, given the discrepancies across the limited research inspecting the iRISA interaction, more targeted efforts are needed to shed light on the factors driving these hypo- versus hyperactivations (e.g., individual differences in performance and treatment status, craving, and task nuances).

### 4.2. Choice and Awareness

Since our previous reviews, continued efforts have also been made to elucidate the neural substrates driving altered decision-making between drug- and non-drug-related stimuli. We developed a pair of picture-viewing choice tasks as lab-simulated measures of drug seeking, which capture effortful and more implicit selection biases toward drug images as compared to pleasant, unpleasant, and neutral images (Moeller et al. 2009). Using this task paradigm, drug-biased choice behavior in SUD versus HC participants has been shown in tobacco (Solinas et al. 2024), cocaine (Moeller et al. 2009, 2018b), methamphetamine (Moeller et al. 2018a), and opioid (McClain et al. 2025, Parikh et al. 2022) use disorders. The direct drug > pleasant choice has informed clinical outcomes such as recent/concurrent (Moeller et al. 2009, 2013) and prospective (Moeller et al. 2013) drug use in those with CUD, as well as opioid-treated chronic pain patients (Moeller et al. 2020a) and treatment dropout in those with HUD (McClain et al. 2025). Interestingly, in people with CUD, a bias for cocaine over neutral-associated monetary choices was observed even when the reward amount was equal or slightly lower for the former, illustrating the maladaptive decisions that are made under cue-reactive states (Strickland et al. 2018). This cocaine-biased choice behavior was partially explained by a greater attentional bias for cocaine cues (measured via eye tracking), and correlated with greater cocaine use severity (Strickland et al. 2018). Using a similar approach, recently detoxified inpatients with severe AUD instead showed avoidance biases for alcohol-related stimuli in a visual probe task, potentially reflecting a high motivation to abstain (Bollen et al. 2021).

Using the choice paradigm in fMRI, heightened choice of cocaine versus food images in treatment-seeking individuals with CUD as compared to HCs was associated with the increased engagement of regions involved in choice difficulty (dorsal ACC) and inhibitory control (supplementary motor area), as well as the dopaminergic ventral tegmental area (Moeller et al. 2018b). These results were interpreted to reflect the increased cognitive conflict and effort monitoring when treatment-seeking people with SUDs process salient drug cues. This study also reported that more negative subject-level cocaine > food preference ratings were associated with higher respective OFC activations, in line with other studies where people with SUDs rate drug cues (including pictures) as unpleasant/negative (Goldstein et al. 2007b, Li et al. 2022) and with the role of the OFC in bidirectional (both positive and aversive) value assignment (Morrison & Salzman 2009, Rudebeck & Murray 2014). The underlying mechanism may be dopaminergic, as suggested by an association between drug > pleasant image choice with higher craving and reduced D2-type dopamine receptor availability in the lateral OFC in people with methamphetamine use disorder (Moeller et al. 2018a). Given a common misconception about the value of drug cues in SUDs, namely their perceived qualification as rewarding, the distinction between reward (connoting pleasant associations with a drug cue) and reinforcement (where behavior can be increased by both pleasant and unpleasant stimuli, in an approach versus avoidance pattern) is important to keep in mind in this context (Koob 2015).

We previously demonstrated a disconnect between self-reported and objectively assessed behavior or brain function among those with SUDs (Goldstein et al. 2007a, Moeller et al. 2016a). Tapping into semantic memory has revealed that a greater tendency among inpatients with cocaine- and alcohol/cocaine-primary SUDs to associate drug-related words with ambiguous images (versus explicitly drug-related or neutral images) is correlated with increased relapse risk and reduced time spent in inpatient treatment (Gómez-Bujedo et al. 2022). We further revealed an impairment in one’s ability to correctly identify their own choice behavior (using the implicit choice task), interpreted as impaired insight (Moeller et al. 2010). In CUD with impaired insight, in addition to reduced gray matter in the rostral ACC, this region’s activity was reduced during Stroop error processing as associated with more frequent recent cocaine use; together, these results were interpreted to suggest blunted PFC-mediated attribution of emotional significance to mistakes, contributing to persistent disadvantageous drug use (Moeller et al. 2014b).

To more directly tap into the neural correlates of impaired self-awareness, we then developed an fMRI task whereby participants responded to statements rating their (and someone else’s) need to change their drug use (and eating behavior). Results showed that individuals who met *Diagnostic and Statistical Manual of Mental Disorders* criteria for CUD fell short of fully recognizing a need for change, as associated with greater drug-seeking behavior (drug versus pleasant image selection on the explicit choice task) (Moeller et al. 2020b). In this study, the medial OFC (part of the rostral ACC/vmPFC) was hyperactive in CUD compared to HCs, especially when evaluating one’s own drug use and as a function of a lower perceived need for change. Together, these results suggest that aberrant engagement of self-referential circuitry (rostral ACC/vmPFC) may be driving these drug-related insight impairments in SUDs (Moeller et al. 2020b). Metacognitive deficits, impaired awareness of one’s own cognition and behavior, may not be specific to drug-related contexts. In individuals with OUD, recent opioid use exacerbated a dissociation between task performance and confidence on a visual perception task with neutral stimuli (Moeller et al. 2024), a pattern also observed in CUD (Moeller et al. 2016a). These deficits were linked to increased cortico-striatal engagement, including greater activation in the ventral striatum and OFC during confidence ratings, particularly when confidence aligned with task performance. These findings highlight metacognition as a potential neurocognitive marker with clinical relevance, such as limiting awareness of maladaptive behaviors and reducing treatment engagement.

### 4.3. Other Cognitive Functions

We previously developed a drug version of the traditional semantic fluency task (Goldstein et al. 2007c). Here we demonstrated that cocaine urine-positive (recent use), but not cocaine urine-negative (abstinence), individuals with CUD generated more drug-related (but not nondrug) words compared to HCs. Correcting for verbal IQ, we later showed that this pattern of drug > neutral fluency correlated with a parallel pattern in the dopaminergic midbrain (in response to pressing the color of drug > neutral words on the emotional Stroop task), suggesting that these drug words carry conditioned incentive properties (Goldstein et al. 2009). Despite their abstinent and treatment-seeking status, the drug fluency behavioral results have now been replicated in inpatients with HUD (McClain et al. 2025). Drug cue–driven narrowing of memory and retrieval could contribute to the superior drug fluency in SUDs. For example, people with AUD showed enhanced memory for neutral scenes associated with alcohol objects as a function of cue salience (and general deficits for those associated with neutral objects)—a pattern that was pronounced in those with more severe drinking patterns (Goldfarb et al. 2020). The underlying mechanisms (e.g., attention bias, memory, retrieval) of the seemingly drug-induced “improvement” of a cognitive function in people with SUDs need to be explored in functional neuroimaging environments. Comparison to nondrug cue processing, and directly between cognitive functions, can offer directionality and specificity to interpretations.

## 5. RECOVERY IN THE NEURAL SUBSTRATES OF iRISA

Neural markers related to abstinence are commonly studied cross-sectionally by comparing current users and abstaining individuals (further stratified by length of abstinence). Fewer studies were performed longitudinally in within-subjects designs to more precisely and dynamically identify biomarkers and track clinical endpoints controlling for group variability. We recently reviewed the abstinence-mediated recovery of brain abnormalities as related to iRISA in people with drug addiction, largely highlighting cortico-limbic functional changes with the sustained cessation of drug use (Parvaz et al. 2022). In this previous review, mixed results suggested the need to better understand the main time frames and trajectories of change (with treatment, abstinence, or reduced drug use) and their underlying neural substrates. Here, we document more recent advances in these efforts and uniquely discuss the neurobehavioral substrates of recovery as mediated by abstinence and neuromodulatory interventions including cognitive-behavioral, pharmacological, and neurostimulation strategies.

### 5.1. Cognitive-Behavioral and Abstinence-Mediated

Cognitive-behavioral interventions and sustained abstinence are candidates for driving recovery-related changes in brain function in SUDs. In this section, we examine how these factors may modulate neurobehavioral markers of recovery, beginning with their impact on cue reactivity.

#### 5.1.1. Cue reactivity.

Baseline drug cue reactivity (and resting-state functional connectivity) is associated with, and predictive of, later drug use outcomes across SUDs. For example, individuals with CUD who showed lower baseline (pretreatment) drug cue reactivity in the ACC, OFC, amygdala, midbrain, and other regions had fewer lapses over 8 weeks of psychosocial outpatient treatment (e.g., drug counseling and active strategies for coping with craving) (Regier et al. 2021). Compared to those who relapsed after 3 months, those who maintained abstinence showed stronger cortico-striatal resting-state functional connectivity (i.e., between the dlPFC and nucleus accumbens) (Berlingeri et al. 2017). Results in methamphetamine use disorder are consistent with the impact of longer-term abstinence (e.g., 10–15 months versus shorter-term abstinence at 1 week to 3 months) on cortical cue reactivity, suggesting a shift in salience attribution from drug to nondrug (sex) reward (Yan et al. 2024). In treatment-seeking smokers, lower baseline (during acute 24-h abstinence compared to satiety) smoking cue–related ACC reactivity was associated with abstinence during a short-term (7 day) quit attempt (Allenby et al. 2019). In treatment-seeking individuals with AUD, lower baseline alcohol cue reactivity (relative to stress cues) in the vmPFC, striatum, and IFG in women, and in the hypothalamus in men, predicted the number (fewer and greater, respectively) of future heavy drinking days during the ensuing 8 weeks of outpatient treatment (Radoman et al. 2024). Similarly, in HUD, lower drug cue reactivity (in the midbrain, caudate, hippocampus, and other areas) was reported in inpatients in protracted abstinence (without medication treatment) as compared to those treated with methadone (Wei et al. 2020). Furthermore, in HUD, stronger resting-state connectivity between key nodes of the salience network (e.g., dorsal ACC), executive control network (e.g., dlPFC), and default mode network (e.g., precuneus) was reported with longer (6–12 month) treatment that included physical rehabilitation and psychological interventions as compared to shorter-term abstinence (7–15 days of detoxification) (Chen et al. 2021). Here, the dlPFC and precuneus connectivity were directly associated with longer abstinence duration (Chen et al. 2021). These patterns are in line with preliminary evidence for resting-state OFC recovery as a function of heroin abstinence (Wang et al. 2021). In our recent longitudinal study mentioned above, we demonstrated that abstinent inpatients with HUD who underwent 15 weeks of treatment that included medications for opioid use disorder and psychosocial interventions (encompassing relapse prevention, seeking safety therapy, and anger management in addition to mindfulness or a support group) showed a reduction in the drug-biased group-level synchronization in the OFC, vmPFC, IFG, dlPFC, and insula when viewing a drug-themed movie; the OFC drug-biased reductions were associated with movie scene–induced craving reductions with treatment (Kronberg et al. 2025).

Together, the pattern of results is mostly consistent: More drug cue reactivity is associated with less abstinence. It is also predictive of relapse yet is amenable for modulation by treatment. Nevertheless, contradictory results have also been reported. For example, greater pretreatment smoking cue–induced reactivity (in the ACC, amygdala, and ventral striatum) predicted longer abstinence following a 9-week smoking cessation program that included counseling and nicotine replacement therapy (Owens et al. 2018). Also, greater drug cue–induced dlPFC activity (and its functional coupling with the thalamus) at baseline predicted craving reductions at a 6-month follow-up in abstinent individuals with HUD after non-medication-assisted treatment (Liu et al. 2021). This variability may be driven by differences in the length of abstinence before the fMRI procedure; abstinence maintenance throughout the study; the detrimental effects of mandatory and non-medication-based detoxification programs such as protracted stress, pain/discomfort, and loneliness/negative affect (Heilig et al. 2010); and other factors inclusive of sex differences.

In addition to standard care and abstinence, mindfulness training such as in the Mindfulness-Oriented Recovery Enhancement (MORE) program, which enhances responsiveness to natural rewards as attributed to the restructuring of hedonic dysregulation, shows promise as a psychosocial intervention in SUDs. Compared to standard treatment, MORE reduced smoking cue reactivity in the insula and amygdala as related to reduced craving in non-treatment-seeking cigarette smokers (Zheng et al. 2024). These patterns are in line with previous results in cigarette smokers where MORE decreased smoking cue reactivity, increasing nondrug savoring–related reactivity, in the striatum and vmPFC (Froeliger et al. 2017). In a related vein, compared to treatment-as-usual in individuals with AUD, cue exposure therapy increased resting-state functional connectivity between the ACC and insula, decreasing ACC-putamen connectivity, suggesting modulation of the salience and habit networks in SUDs (Farré-Colomés et al. 2024). This result is consistent with earlier research where cue exposure therapy reduced drug cue reactivity in the insula, other limbic areas, and the PFC in AUD (Vollstädt-Klein et al. 2012). Other promising interventions, inclusive of motivational interviewing (Feldstein Ewing et al. 2011) and neurofeedback training (Kirsch et al. 2016), remain to be explored vis-à-vis their impact on reducing neural cue reactivity in SUDs.

#### 5.1.2. Inhibitory control.

Only a few clinical neuroimaging studies targeted the potential functional recovery of inhibitory control as associated with abstinence and/or cognitive-behavioral treatments in SUDs. In our recent study, we reported the recovery of inhibitory control–related anterior PFC and dlPFC engagement in individuals with HUD after continued inpatient treatment (that encompassed MORE versus support therapy in the context of standard treatment, as described above); increases in anterior PFC engagement were associated with increases in target detection sensitivity (*d*^′^) on the stop-signal task (Ceceli et al. 2024). Similarly, inhibitory control– related IFG activation on the stop-signal task was higher during abstinence compared to satiety in smokers (Chaarani et al. 2019). Further, compared to smokers who relapsed, smokers who maintained abstinence throughout a 12-week smoking cessation trial exhibited anterior insula hyperactivations during smoking cue–related no-go trials (Gilman et al. 2018). In contrast, increased inhibitory control–related activity in the IFG during abstinence (coupled with its weaker task-based connectivity with the thalamus and its lower gray matter volume) correlated with earlier smoking in a laboratory-based smoking relapse analog task (Bell & Froeliger 2021, Froeliger et al. 2017). More such studies are clearly needed.

### 5.2. Pharmacological

Pharmacological interventions may offer promising avenues for modulating the neural correlates of addiction, including those implicated in the iRISA model. In this section, we highlight recent evidence on how specific compounds may influence cue reactivity and inhibitory control processes across SUDs.

#### 5.2.1. Cue reactivity.

Naltrexone, an opioid antagonist that primarily targets the MOR, is commonly used for the treatment of opioid use disorder and AUD. Mechanistically, naltrexone downregulates drug-induced dopamine release to block the rewarding effects of drug use, especially via the dopaminergically innervated ventral striatum (Benjamin et al. 1993). Accordingly, the extended release of naltrexone reduces ventral striatal and OFC reactivity to opioid versus aversive or sexual cues in individuals with opioid use disorder, with reductions in OFC tracking decreases in withdrawal symptoms (Shi et al. 2018). However, the lion’s share of the evidence for naltrexone’s effects on drug cue reactivity comprises clinical trials that target AUD, with largely consistent patterns indicative of a reduction of striatal alcohol cue reactivity and improvements in alcohol use outcomes. For instance, in inpatient men, putamen drug cue reactivity decreased when AUD treatment was supplemented with naltrexone as associated with reductions in alcohol craving (consistent with craving incubation, increased cue reactivity was otherwise observed) (Bach et al. 2020). Here, while higher putamen cue reactivity was associated with quicker relapse to heavy drinking, with naltrexone those with high cue reactivity at baseline had a longer time to relapse (Bach et al. 2020). These effects were also evident in the ventral striatum (Bach et al. 2021), directly replicating a previous demonstration in AUD of the interaction between baseline ventral striatal cue reactivity and naltrexone treatment on the latency to relapse (Mann et al. 2014), observed also in non-treatment-seeking populations (Lim et al. 2020).

Another promising agent is the neuroimmune modulator ibudilast. Compared to placebo, ibudilast attenuates the ventral striatal signal during the passive viewing of alcohol (versus other beverage) cues in non-treatment-seeking individuals with AUD, as associated with fewer prospective (1 week later) drinks per drinking day (Grodin et al. 2021). These striatal alcohol cue–reactivity reductions may be attributed to ibudilast’s phosphodiesterase-4 and phosphodiesterase-10 inhibitory profile. These enzymes are involved in proinflammatory cytokine expression (Page & Spina 2011) observed abundantly in the striatum (Nishi et al. 2008), with downregulatory effects on dopaminergic signaling via cyclic adenosine monophosphate–dependent protein kinase A (Ramirez & Smith 2014). Interestingly, ibudilast also reduces alcohol (versus nonalcohol beverage) cue reactivity in the OFC, IFG, and dlPFC (in addition to the dorsal striatum) and drinks per drinking day in individuals with higher baseline C-reactive protein levels (indicative of elevated inflammation), suggesting that its effects are pronounced in those who need it most (Grodin et al. 2023). Ibudilast also reduced functional connectivity between the ventral (and dorsal) striatum and OFC/ACC during alcohol compared to nonalcohol beverage passive viewing, as associated with fewer drinks per drinking day (Burnette et al. 2021). These PFC effects may similarly be driven by phosphodiesterase, as these enzymes are also expressed in the ACC and other PFC regions (Pérez-Torres et al. 2000).

Mesocorticolimbic functional decreases during cue reactivity in AUD are also illustrated using pharmacological agents that act on gamma-aminobutyric acid (GABA) neurotransmission, particularly in a dose-dependent manner. At higher doses (75 to 270 mg per day), baclofen, a selective GABA_B_ receptor agonist (primarily used as a muscle relaxer), reduced ventral tegmental area, amygdala, OFC, and ACC activity (and the functional connectivity between the ventral tegmental area and these cortical regions) during alcohol cue exposure as compared to placebo in AUD (Beck et al. 2018). These higher doses were also associated with a higher likelihood of maintaining abstinence from alcohol use (Beck et al. 2018) and fewer prospective heavy drinking days (Logge et al. 2021). In contrast, low-dose baclofen increased the engagement of the dlPFC and ACC during alcohol cue exposure as compared to placebo that nevertheless was associated with a reduced likelihood of early relapse in people with AUD (Holla et al. 2018), warranting further investigations that control for dosage. Mesolimbic drug cue reactivity is also reduced with cannabidiol, a non-intoxicating component of cannabis and an allosteric modulator of the GABA_A_ receptor, which potentially underlies its anxiolytic and anticonvulsant properties (Bakas et al. 2017). For example, a single-dose (800 mg) cannabidiol challenge dampened alcohol cue reactivity in the ventral striatum, as well as cue-induced craving, as associated with higher plasma cannabidiol concentration (Zimmermann et al. 2025), suggestive of dose effects. Glucagon-like peptide-1 agonists also modulate GABAA receptors (Korol et al. 2015), through which they reduce alcohol intake in rodents (Chuong et al. 2023). In people with AUD, the glucagon-like peptide-1 agonist exenatide reduced alcohol cue reactivity in the ventral striatum with no effects on drinking frequency (Klausen et al. 2022), warranting further fMRI investigations of this compound’s effects in human SUDs.

The recent efforts to pharmacologically modulate neural cue reactivity in drug addiction have largely been exerted toward AUD, with very limited reports (or none to date) in tobacco, cannabis, cocaine, and opioid use disorders. Cocaine addiction is especially of concern, as no US Food and Drug Administration–approved pharmacological CUD interventions currently exist. Previously, we demonstrated beneficial effects of methylphenidate (a partial dopamine agonist) during the color-word and drug Stroop tasks (with normalizations in the dlPFC in the former, and ACC and OFC function during drug cue processing in the latter) and resting-state functional connectivity (notably between the ventral striatum and OFC) in individuals with CUD (Goldstein et al. 2010; Konova et al. 2013; Moeller et al. 2014a, 2016b). More recently, in people with CUD, we revealed that a single-dose methylphenidate challenge administered during memory reconsolidation (i.e., a time-dependent window during which a consolidated memory is rendered labile for alteration) modulates drug cue extinction-related vmPFC overreliance (and its mesolimbic connectivity) (Ceceli et al. 2025). Here, we also found effects extending to lab-simulated drug seeking, whereby only the aberrant vmPFC signaling under placebo correlated with drug-biased viewing choices the next day (Ceceli et al. 2025). Such drug repurposing, especially for enhancing cognitive function, should also be tested in the other SUDs. Another example is oxytocin, which reduces dorsomedial PFC drug cue reactivity in individuals with CUD, with sex-specific effects (men > women) observed in the amygdala in those with a history of childhood trauma ( Joseph et al. 2020). More recent efforts have targeted glutamatergic modulation with little success in cannabis use disorder and CUD. For example, gabapentin, a glutamate and GABA modulator that is primarily used as an anticonvulsant, increased glutamate in the dorsal ACC and putamen in cannabis use disorder; however, it increased drug cue reactivity in the midcingulate cortex, and effects were moderated by randomization order and cigarette smoking status (Prisciandaro et al. 2022). Similarly, N-acetyl cysteine, which metabolizes into l-cysteine, a precursor to glutathione that can induce agonist-like effects on glutamate receptors (Atkuri et al. 2007, Sedlak et al. 2019), has not yielded the expected significant drug cue reactivity reductions in people with CUD (Schulte et al. 2019).

#### 5.2.2. Inhibitory control.

Naltrexone increases OFC activity during a go/no-go task in abstinent individuals with AUD; in the absence of behavioral group or treatment differences, and with more days of abstinence tracking higher baseline inhibitory control PFC activity (across treatments), this pattern suggested a beneficial neurocognitive adaptation (Nestor et al. 2019). Given OFC’s role in regulating inhibitory control processes (Brockett & Roesch 2021), and its abundant expression of MOR (Mansour et al. 1995) [which naltrexone targets (Niciu & Arias 2013)], naltrexone may be enhancing a compensatory mechanism to normalize inhibitory control. Nalmefene, another opioid antagonist (targeting MOR, among other receptors), has also been used for treating AUD. Here, nalmefene increased resting-state functional connectivity between the ACC and OFC/dlPFC, while decreasing nucleus accumbens and insular coupling with the PFC (Grundinger et al. 2022). These results were interpreted to show nalmefene’s effect on improving communication between nodes that regulate conflict monitoring (ACC) and cognitive control (OFC and dlPFC), while dampening cortico-insular-striatal coupling that may impact the interoception of craving.

Given the association between noradrenergic neurotransmission and response inhibition via the PFC (Chamberlain et al. 2006), recent research has leveraged selective norepinephrine (and serotonin) reuptake inhibitors, commonly used to treat depression, as therapeutic agents for normalizing inhibitory control brain function in SUDs. For example, under atomoxetine, a selective noradrenaline reuptake inhibitor, modest improvements in stopping performance (i.e., quicker stop-signal reaction times) in individuals with CUD were associated with IFG activity increases during inhibitory control on the stop-signal task (Zhukovsky et al. 2022). These IFG activations were predicted by worse baseline task performance and higher plasma atomoxetine concentration, suggesting that those who are most affected by inhibitory control deficiencies also benefit most from the atomoxetine intervention, in a dose-dependent fashion (Zhukovsky et al. 2022). The same atomoxetine challenge in individuals with CUD was not as effective during the flanker task, only modulating brain network connectivity during congruent trials, suggesting that atomoxetine-mediated normalizations in brain function may be more specific to attentional processes (Nestor et al. 2024), at least in CUD.

The variability in therapeutic efficacy across these studies may be related not only to nuances in the cognitive tasks used (stop-signal task versus flanker), but also to pharmacogenetic heterogeneities in neurotransmission. Mirtazapine, a selective 5HT2A/2C serotonin receptor antagonist, which has been shown to suppress cocaine seeking preclinically (Barbosa-Méndez et al. 2017), decreased cocaine craving in abstinent individuals with CUD (Nanni-Alvarado et al. 2022), potentially via reducing an aberrant ACC-hippocampal effective connectivity during the drug Stroop task (Ma et al. 2018, 2022). Importantly, this mirtazapine-mediated effect was less evident in those carrying the single nucleotide polymorphism variant of the serotonergic receptor [which is associated with hypoactive serotonergic function (Land et al. 2019)] as compared to the wild-type receptor profile (Ma et al. 2022), suggesting a specific genetic descriptor for the optimal pharmacological mitigation of drug-biased processing in CUD.

### 5.3. Neurostimulation

The iRISA model proposes dysfunction in specific anatomically defined brain regions, suggesting that their modulation by spatially targeted tools can improve treatment outcomes with high functional specificity. Electromagnetic brain stimulation tools have developed rapidly in the last two decades, and a growing body of literature suggests that they can modify the cortico-striatal circuitry to improve addiction outcomes, including reducing drug craving and consumption (Mehta et al. 2024). Here we focus on transcranial direct current stimulation (tDCS), transcranial magnetic stimulation (TMS), and deep brain stimulation, although we recognize that additional promising tools are being developed [e.g., transcranial focused ultrasound (Rezai et al. 2025)].

The noninvasive techniques (tDCS and TMS) have focused predominantly on targeting the dlPFC due to its proximity to the scalp and its well-established dysfunction in drug addiction (Mehta et al. 2024), collectively showing moderate effects on reducing craving and drug consumption across SUDs. The vmPFC and frontal pole have also been successfully targeted (Soleimani et al. 2024). For example, a series of studies have demonstrated reduced cortico-striatal drug cue reactivity, associated with reduced craving, in response to TMS targeting the vmPFC in CUD (Kearney-Ramos et al. 2018) and AUD (McCalley et al. 2023). Studies assessing inhibitory control effects of brain stimulation have been less common, yet TMS targeting the right IFG improved inhibitory control–related functional connectivity, reducing craving and smoking, in cigarette smokers (Upton et al. 2023). Using neurostimulation to enhance inhibitory control (and other cognitive functions) under drug cue reactivity in SUDs is a future direction to be pursued. Deep brain stimulation studies have predominantly implanted electrodes in the nucleus accumbens and have similarly reported improvements in craving and abstinence, although sample sizes are limited due to the invasive nature of this neurosurgical technique (Zammit Dimech et al. 2024). Importantly, in stimulating a given brain region, second-order effects on synaptically connected networks have to be considered or even targeted. For example, via synaptic connections from cortical regions that are easier to noninvasively stimulate, e.g., the vmPFC, itis possible to impact subcortical regions that are difficult to reach, e.g., the striatum or amygdala (Soleimani et al. 2024, 2025). In addition to considering brain stimulation as a tool for enhancing recovery, we can also view it as a causal manipulator of the cortico-striatal circuitry. The observed improvements in drug craving and use therefore also serve as causal evidence for the role of these brain regions in these select addiction phenotypes.

Collectively, these early results suggest that cortico-striatal brain networks can be targeted with electromagnetic brain stimulation to facilitate recovery in SUDs. Despite these promising early results, there is a great deal of variability in responses to brain stimulation, likely with a large contribution from variable stimulation protocols, study designs, and type of SUD targeted. The precision and reliability of these methods will likely improve going forward, particularly as individually tailored anatomically and brain-state guided approaches become increasingly adopted and standardized (Soleimani et al. 2024). Future studies can also explore the combination of these approaches, and brain targets, toward enhancing recovery in SUDs. For example, a remote self-administered tDCS trial following a TMS trial could be tested for a long-term sustained effect whereby intervention is available on command/as needed. Further, targeting of dlPFC/IFG versus vmPFC functions does not have to be mutually exclusive; rather the decision can be based on individual (e.g., urgency of the specific dysfunction) and phase (e.g., abstinence or craving) factors. Indeed, network synchronization can be promoted by high-definition, multisite, focal alternating current stimulation (Helfrich et al. 2014).

## 6. NOVEL DIRECTIONS

So far, we have commented on the characterization and recovery of drug addiction pathophysiology from the lens of the iRISA model, focusing on traditional neuropsychological and cognitive neuroscience methods. These well-validated tasks translate complex real-world phenomena into well-controlled laboratory measures (e.g., presenting static drug and nondrug images to examine the neural signature of cue reactivity and drug seeking). However, in the real world, drug addiction is characterized by rich and dynamic drug-biased experiences to which the constrained lab-derived tasks may not be sufficiently sensitive. Recent trends have therefore adopted more naturalistic approaches with complex multidimensional stimuli and computational methods to model drug addiction. For example, studies leveraging modern natural language processing, particularly those using recent large language models, have successfully predicted treatment outcomes from patient spontaneous speech (Agurto et al. 2025) and social media (Curtis et al. 2023, Kornfield et al. 2018, Liu et al. 2022), uncovering linguistic features of complex cognitive and emotional processes associated with relapse and abstinence. In parallel, 24/7 wearable devices and ecological momentary assessments dynamically and in real time track rest-activity rhythms (e.g., sleep-wake cycles), craving, and drug use, offering scalable insights into risk and vulnerability prediction, crucial for the development of personalized interventions. Complementing these efforts, a naturalistic brain imaging approach, using movies to examine neural responses to the competition of drug cues with other reinforcers within emotionally engaging narratives, captures salience attribution and treatment effects in ways that picture-based static tasks may miss (Kronberg et al. 2025). These approaches are being further enriched by computational models grounded in reinforcement learning and value-based decision-making, which help bridge between neuropsychological functions (e.g., value tracking and risk tolerance) and link them to clinical outcomes (Konova et al. 2020). Together, these novel directions reflect a shift toward more real-life dynamic and integrative methods to improve the ecological validity and clinical applicability of addiction neurobiology research, as we discuss in more detail in the Supplemental Appendix.

## 7. CONCLUSIONS

Recent advances in drug addiction neuroimaging continue to support the tenets of the iRISA model. The reviewed evidence across substances largely underscores cortico-striatal hyperactivations to drug cues, even during the downregulation of this cue reactivity via reappraisal. This drug-biased salience attribution, recently measured as a synchronous group pattern during naturalistic movie watching, is further associated with craving, medication dosage, history of withdrawal, and other individual differences such as sex and hormonal profiles. Neuroimaging studies in drug addiction also corroborate PFC and basal ganglia regional and network-level hypoactivations and altered connectivity during inhibitory control, also driven by individual (lifetime) differences encompassing severity of drug use, aging, and trauma history. To behaviorally probe the iRISA-related interaction (between cognitive function and salience attribution), we have integrated drug cues (versus neutral but, importantly, also other salient cues) into tasks tapping into higher-order cognitive functions, modifying into drug-related versions the Stroop task, stop-signal task, semantic fluency, and choice tasks. Additional similar efforts have targeted attention, feedback learning (and other learning and memory functions), decision-making, and semantic processing, suggesting drug cue–induced dysfunction in cortico-striatal networks (inclusive of the salience/value networks encompassing the ACC and OFC regions) in people with SUDs. Here, the conditioned responses to drug cues are leveraged as potent modulators of the select cognitive function, amenable for objective measurement (e.g., expressed as an attentional bias, physiological arousal, distinct neural patterns) and commonly associated with drug use severity and predictive of future outcomes including relapse. Discrepancies across studies are evident (e.g., hyper- or hypoactivations during the iRISA interaction, impaired behavioral performance or its enhancement by a drug context, resting-state functional hyper- or hypoconnectivity patterns vis-à-vis clinical measures), and targeted continued efforts are needed, with an eye toward the importance of variability in task paradigms but also covariates inclusive of treatment status, substances used, and behavioral performance. The cognitive functions that show relatively intact and/or less malleability to the impact of drug cue reactivity remain to be identified, of importance for the mechanistic study of double dissociations and potential intervention development. Importantly, overlaying these impairments are deficits in behavioral insight and self-awareness that can be further subsumed under metacognitive deficits, together thought to be driven by rostral ACC/vmPFC and insula dysfunction and modulated by the recency of drug use.

Emerging evidence from more naturalistic measures such as spontaneous speech and rest-activity rhythms supports their utility as sensitive, ecologically valid, and affordable behavioral markers. These markers can be continuously and (semi)automatically captured to access dynamic real-world behavioral patterns to support scalable dense-sampling efforts. An important next step is the identification of markers that inform functional and structural underpinnings of addiction pathophysiology, with the goal of monitoring and predicting treatment outcomes. Extending this approach to additional modalities such as the use of full-length movies, immersive environments, additional language-based tasks, and digitally recorded data from smartphones or wearable technology, while targeting addiction-related processes (e.g., cue reactivity and its regulation), could help resolve inconsistencies in the literature. Such efforts may clarify how signs and symptoms of SUDs serve as proxies for brain function, with implications for neuroscience, treatment development, and public health policy inclusive of timely risk identification and prevention.

The cortico-striatal hyperactivations during drug cue reactivity and largely hypoactivations during inhibitory control are normalized with abstinence, pharmacological agents [including naltrexone, ibudilast, and GABA modulators (primarily documented for cue reactivity in AUD)], and other modulators that target noradrenaline and serotonergic receptors (showing promise for attenuating cue reactivity in CUD), as well as neurostimulation-based interventions. Limited evidence for neural recovery with cognitive and behavioral interventions (e.g., cue exposure, motivational interviewing, mindfulness) calls for additional studies; other promising modalities (e.g., neurofeedback) remain to be explored. Given the complex nature of SUDs (driven by differences attributed to the substances used, comorbidities, recency and/or severity of use, treatment received, and a myriad of other factors), it is safe to postulate that a combination of modalities could lead to the development of the most effective and efficient interventions, timely delivered (e.g., at particular phases of the addiction cycle and/or menstrual cycle) for enhanced individual tailoring.

Missing from this review are studies conducted in adolescents and/or children or other populations of interest/relevance (e.g., at risk for SUDs), needed for the identification of premorbid and vulnerability factors, a goal for a future in-depth review. Further, we focused on fMRI studies of iRISA in SUDs, yet underlying traits (e.g., impulsivity) (Belin et al. 2016) are common to numerous comorbid disease processes as expressed throughout the body and lifespan. The commonalities/differences with these other diseases would also be important to explore in future efforts. Powerful computational tools should be employed for identifying patterns that uniquely characterize the pathophysiology of SUDs, with direct comparisons across different drugs of abuse and potentially as relevant to behavioral addictions, used also for tracking the divergence of longitudinal trajectories of change and for predicting clinical endpoints.

Among the cognitive functions, this review focused on inhibitory control, although other higher-order executive functions were mentioned. Additional studies are needed to explore interrelated self-awareness, insight, and metacognitive functions as well as their potential association with emotional impairments [e.g., facial emotion recognition (Rabin et al. 2022)] and broader social processing difficulties in drug addiction. In this vein, the presence or absence of others may play a critical role in the risk of and resilience to SUDs [e.g., childhood neglect or other trauma (Cicchetti & Handley 2019) and adulthood loneliness as risk factors (Hosseinbor et al. 2014)]. Empirical investigations can explore social influences such as peers who can not only predispose toward but also protect against drug use, which is of relevance to recovery mediated by Alcoholics Anonymous/Narcotics Anonymous meetings and other types of treatment (e.g., individual or group therapy, inpatient or outpatient treatment).

Considering the historical emphasis on subcortical regions and networks, the PFC higher-order executive cognitive functions and the power of a drug-relevant context to subvert them in addiction remain relatively underexplored. This review sheds light on the importance of the neural correlates of iRISA in humans with SUDs, providing support for this model using recent neuroimaging studies. The next decade of research will undoubtedly see an increased use of artificial intelligence and large language models, big datasets, and naturalistic approaches to recording brain function and behavior in people with SUDs for the purposes of enhancing mechanistic understanding, predicting and preempting risky situations, and enhancing treatment and well-being in these devastating disorders.

## Supplementary Material

Supplementary material
