# Supplementary material for: The Impaired Response Inhibition and Salience Attribution Model of Drug Addiction: Recent Neuroimaging Evidence and Future Directions
Source: Annu Rev Psychol. Author manuscript; Available in PMC 2026 Apr 21. (PMC13099233; doi:10.1146/annurev-psych-040725-025923)
Supplement: Supplementary material [file NIHMS2162545-supplement-Supplementary_material.pdf]

The impaired Response Inhibition and Salience Attribution (iRISA) model of drug addiction: recent neuroimaging evidence and future directions

Ahmet O. Ceceli, Yuefeng Huang, Greg Kronberg, Natalie McClain, Sarah G. King, Eduardo R. Butelman, Nelly Alia-Klein, & Rita Z. Goldstein

Supplemental Appendix

Novel directions in addiction research

a. Naturalistic behavior

Recent innovations in language modeling and transformer-based large language models (LLMs) have accelerated promising applications of natural language processing for semi-automating medical tasks like screening, diagnosis, and outcome prediction. Language is ubiquitous in both clinical (e.g., electronic health records, clinician-patient interactions) and non-clinical (e.g., everyday interpersonal communication, social media) settings, providing an easily accessible and versatile target for ecologically relevant discoveries. It is the core medium with which clinicians diagnose and assess patients with substance use disorders (SUD) and through which recovering individuals receive care in the form of counseling and community/peer support.

Only a few studies to date have assessed language as a measure of addiction-related cognitive processes, with several of these focusing on outcome prediction in treatment-seeking populations. In a recent study, semantic features from an open-ended speech task, in which initially abstinent individuals with cocaine use disorders (CUD) spoke about their personal experiences with drug use and abstinence, predicted outcome measures including craving and withdrawal symptoms, abstinence length, and past-90-day cocaine use (Agurto et al. 2025). Specifically, a predictive model built on the semantic similarity between participants' speech and items from surveys assessing craving, anxiety and quality of life outperformed models using only demographics or standard addiction survey scores (inclusive of the predicted drug use variables assessed at baseline). This model used sentence embeddings extracted with a pre-trained Bidirectional Encoder Representation from Transformers (BERT) model (Reimers & Gurevych 2019), and successfully predicted drug use severity measures obtained 12 months later (Agurto et al. 2025).

Studies have also demonstrated substance use and treatment outcome prediction using social media language. In a retrospective study on Facebook posts during the previous two years from 269 individuals in outpatient treatment for SUD (primarily to cannabis, cocaine, and heroin), BERT embeddings were used to predict treatment retention and relapse in the first 90 days of treatment, outperforming a standard clinical intake interview (the Addiction Severity Index) (Curtis et al. 2023). Using a topic modeling approach, another study from the same group that included 206 outpatients identified specific linguistic features in Facebook posts that were associated with treatment retention for at least 90 days (Liu et al. 2022). These included topics defined by social/affiliative contexts (family, religion), positive emotional expression, and use of first-person pronouns. A similar topic modeling analysis of posts from an alcohol use disorder (AUD) recovery discussion forum, including 104 individuals who had recently completed an inpatient treatment program, identified topics associated with abstinence 12 months later (Kornfield et al. 2018). In this study, language hypothesized to reflect higher-order cognitive mechanisms was negatively correlated with relapse, whereas words related to inhibitory actions and negative emotions correlated positively with relapse. In contrast to (Liu et al. 2022), topics defined by

Downloaded from www.annualreviews.org. Guest (guest) IP: 100.35.127.123 On: Mon, 13 Apr 2026 18:20:00

social/affiliative contexts or the use of first-person pronouns did not contribute significantly to outcome prediction in this earlier study (Kornfield et al. 2018).

Another advantage of automated natural language processing methods is their capacity for handling massive datasets that are generally unattainable in laboratory settings. In this way, hypotheses derived from smaller, focused studies in specific patient populations can be tested on a much larger scale with spontaneously generated, real-world naturalistic language data that is freely accessible in topical forums on social media platforms such as Reddit (Valdez & Patterson 2022). For example, one recent study on 2 million Reddit posts identified the characteristic emotional profiles in language from forums for tobacco, alcohol, and cannabis use cessation, with expressions of both negative (anxiety, disgust) and positive (gratitude, pride) emotions featuring prominently (and changing with abstinence length) (Yang et al. 2023). A caveat of such studies is the anonymous nature of the data, which includes posts from unknown/unverified individuals and sources. However, the content of these forums is generally consistent with established addiction recovery programs (Valdez & Patterson 2022), suggesting such data can be useful for understanding naturalistic behaviors and social/emotional dynamics in individuals seeking social support in the real-world.

In addition to language-based methods, innovations in wearable device technologies and phone applications have expanded naturalistic approaches and provide affordable, ecologically valid measures for assessing the severity and risk of SUD. For example, individuals with SUD frequently exhibit disrupted circadian rhythms (Conroy et al. 2012). Indeed, using actigraphy, measured via continuous accelerometer wear, rest-activity rhythms (i.e., the sleep-wake cycle) were most disrupted in individuals with heroin use disorder (HUD) receiving medications for opioid use disorder, compared to those without medication and healthy controls (HC), and as associated with longer years of heroin use and lower daytime light exposure (Zhang et al. 2025). Furthermore, greater sleep-wake irregularity was correlated with a lower prevalence of default mode network-related brain states in HUD, aligning with previous findings on this network's impairments in individuals with SUD (Zhang & Volkow 2019). Importantly, these disruptions in rest-activity rhythms were associated with dopamine receptor availability in the dorsal and ventral striatum, linking circadian rhythm disturbances to a core neurobiological mechanism in drug addiction (Zhang et al. 2021). Together, these results could be used to develop individually tailored timely delivered interventions, such as increasing light exposure or physical activity and exercise (Greenwood 2019), the latter known to lead to improved treatment outcomes (Morais et al. 2018), potentially in combination with relevant pharmacological interventions.

Using these wearable devices and phone applications, drug use (e.g., type, amount, frequency, craving, and other severity measures) can be tracked with ecological momentary assessments, or experience sampling, where data is gathered via real-time reports of momentary experiences during everyday life, combining event-based reports of substance use and notable occurrences including stressful events with time-based assessments (Shiffman 2009a,b). These dense measures in the real-world can make it uniquely possible to examine time-lagged associations between fluctuating clinical events with select behavioral (e.g., speech, rest-activity rhythms) and other (e.g., brain) variables, ultimately allowing the identification of risky drug use situations in real-time. As part of these measures one could assess core addiction substrates [e.g., drug wanting more than liking with the Sensitivity to Reinforcement of Addictive and other Primary Rewards (STRAP-R) (Goldstein et al. 2010) and habit vs. goal driven behavior (e.g., via the two-step decision task) (Daw et al. 2006)] for their modulation [e.g., to preempt the incubation of craving (Grimm et al. 2001; Parvaz et al. 2016)].

Downloaded from www.annualreviews.org. Guest (guest) IP: 100.35.127.123 On: Mon, 13 Apr 2026 18:20:00

b. Naturalistic brain imaging

Movies have emerged as increasingly popular "naturalistic" stimuli in neuroscience, mimicking natural experience in that they are dynamic, multimodal (i.e., auditory and visual), and embedded in narrative context (Sonkusare et al. 2019). Movies are also designed to be maximally engaging and evocative of emotional and cognitive processes relevant for psychiatry (Eickhoff et al. 2020). Improving ecological validity, movie stimuli therefore can be a rich testbed for the predictions of the iRISA model.

Previous work has assessed the drug-biased salience attribution in addiction by averaging responses to different stimulus categories (e.g., pictures) in isolation and comparing groups or different stimuli on the magnitude of averaged responses (Huang et al. 2024). While this approach has excellent interpretability and statistical power, it misses several key aspects of the structure of natural salience attribution. For example, in natural experience competition for salience occurs directly between different stimuli in our immediate sensory inputs. Moreover, this competition occurs dynamically in a hierarchy of timescales such that faster fluctuating stimuli are embedded in and constrained by slower developing contextual information (Hasson 2025). This dynamic, multi-timescale, and context dependent competition for salience attribution is essential for interpreting a movie and navigating the real-world, but it is entirely missing in standard picture-based fMRI tasks.

While movies have been used to study several psychiatric or neurological conditions (Eickhoff et al. 2020), they have only recently been applied to the study of drug addiction. In a study mentioned in this review, where inpatients with HUD watched the first 17 minutes of "Trainspotting", an Oscar nominated movie about HUD, only nine of the scenes included highly salient depictions of drug use and sequelae, while 15 scenes included depictions of other reinforcers (food, social scenes) (Kronberg et al. 2025). Yet, the drug related hyper-reactivity was robust; also, OFC normalizations (compared to HC) after three months of inpatient treatment (which correlated with concomitant reductions in craving, and interpreted to reflect recovery with treatment) were not observed with a picture-based task performed by the same patients (Huang et al. 2024). This initial study suggests that measures of neural function during processing of naturalistic stimuli that portray core SUD experiences provide sensitive markers of disease phenomenology and treatment effects.

An important and simple way to build upon this work is to apply a similar approach to other movies, contributing to a database of dynamic naturalistic drug cue reactivity. This database will be crucial for testing generalizability of results. While our initial study analyzed salience attribution at the fastest possible resolution for fMRI (single TR), future work can also compare responses at slower timescales and larger contextual scopes within a given movie, but also between movies (i.e., the largest contextual scope). As mentioned above, previous work in movie fMRI indicated that contextual information is integrated at varying timescales throughout the brain (Hasson et al. 2008), suggesting that different brain networks may be involved in salience attribution at slower timescales. How addiction-related impairments in salience attribution vary by timescale is an important question for future work, as addiction can be framed as narrowing of the temporal scope of an individual's goals and cognition (Bickel et al. 2007).

Given the rich emotional and cognitive demands of engaging movies, they can also be a potent platform for testing the iRISA interaction between drug cue reactivity and PFC-based cognitive function. There is a methodological challenge here in measuring cognition during a movie without disrupting the immersive experience of the movie. One interesting approach would be to use localizer tasks to determine individualized brain activations for a given cognitive task, then look for their reactivation during a movie. An alternative could be to incorporate minimally disruptive button presses to record behavior during the movie, e.g., a modified Stroop-like task where subjects press a button in response to border color [analogous to word color in the drug

Downloaded from www.annualreviews.org. Guest (guest) IP: 100.35.127.123 On: Mon, 13 Apr 2026 18:20:00

Stroop task (Goldstein et al. 2007)], where we would expect unique performance during drug scenes in people with SUD.

A major challenge of embracing complex naturalistic stimuli is that these tasks are by definition less constrained, leading to challenges in interpreting results. Human annotation of naturalistic stimuli can be used, but there could also be a major role for LLMs, and similar multimodal transformer models, which can embed text, images, audio, and video in an interpretable vector space (Gemini Team Google et al. 2024). These models are remarkably effective at handling complex real-world and large data and can provide powerful tools for quantitative analyses of naturalistic stimuli and data (inclusive of neuroimaging). We therefore expect the use of LLM-based analysis and predictive modeling to develop alongside naturalistic neuroimaging datasets in addiction (and psychiatry more broadly). Advances in these models are being made rapidly, particularly with respect to video content. Interestingly, as of writing this review, models that can interpret and generate videos are beginning to mature with clear applications to movie neuroimaging (Abi-Dargham et al. 2023). There is the potential in the future to use these models to generate naturalistic stimuli that are tailor-made for specific research questions (e.g., comparisons between HUD and CUD and other SUD types and between early vs. later abstinence in search for risk vs. resilience markers).

c. Insights from computational psychiatry

On the route to develop, test and improve clinical interventions and other applications, an important future direction is to translate iRISA into computational models as a tool for enhancing mechanistic understanding, handling complex datasets and developing sensitive, specific, and generalizable biomarkers. While decades of research have provided tremendous fundamental insight, clinical neuroimaging biomarker development in drug addiction is still in very early stages. Pursuing models that predict addiction severity and treatment outcome via cross-validated machine learning is a promising path forward (Yip et al. 2020). Crucially, this approach is well-suited to handle complex real-world data and prioritize generalization to unseen samples, a necessary biomarker feature. This approach is becoming increasingly common, with several recent papers showing promising results (Agurto et al. 2025; Koban et al. 2023; Yip et al. 2019). Although data-driven in the sense that they learn patterns from high dimensional data, there is still a large role for mechanistic theories in selecting and refining the inputs to these models and in results interpretation. That is, the better the diagnostic signal/noise ratio in the inputs and the more we can remove irrelevant dimensions, the more likely machine learning algorithms are to find useful patterns (e.g., for biomarker development). The evidence described throughout this review suggests specific behaviors, neuroimaging tasks, statistical contrasts, and brain networks that should be discriminative in diagnosing and characterizing addiction severity. Which of these features are most discriminative and how they might be combined to maximize sensitivity and specificity in held out samples is an open question that should be the subject of an iterative refinement process in the coming decade.

Given the recurrent and cyclical nature of drug addiction and high rates of relapse and treatment dropout, especially in opioid use disorder (Hser et al. 2014), a key translational goal is to monitor individuals longitudinally. Here, computational tools may be of particular interest as they can help differentiate individuals who are at greater risk or vulnerability vs. those demonstrating resilience during treatment. A promising candidate neuropsychological process for such prediction purposes is value-based decision-making where formal theory-driven computational models can help quantify specific (inclusive of behavioral) subcomponents that are more precisely related to clinical measures (e.g., severity of disorder, relapse). Here algorithmic reinforcement learning (RL) models have been highly influential, with components of these models mapping readily onto the iRISA framework (Gueguen et al. 2021). In a recent demonstration of this approach, we used an RL model to document blunted dopaminergic reward prediction error signaling (in the ventral striatum) in CUD (Konova et al. 2023). An

Downloaded from www.annualreviews.org. Guest (guest) IP: 100.35.127.123 On: Mon, 13 Apr 2026 18:20:00

RL model was also used to document insensitivity of reward values to context in recently abstinent individuals with opioid use disorders but not in those at later abstinence (>3 months) or HC, with value adaptation correlating with continuous abstinence and subjective craving (Gueguen et al. 2024). Of mention is another naturalistic study that used RL modeling to show that a mindfulness intervention reduced smoking by specifically influencing the expected future value of smoking behavior (Taylor et al. 2022).

In addition to RL and value-based decision-making, computational models of risk detection and tolerance have shown promise in identifying component processes related to clinical outcomes in addiction. Using a clever design and computational modeling of risky decision-making to separate individuals' tolerance for known vs. unknown risk, a study in opioid use disorder exemplifies this approach, showing that the tolerance for unknown risk predicted relapse at a following visit (Konova et al. 2019). Inferences about addiction related behaviors are also improved by computational modeling in AUD, as risk taking propensity (measured by the balloon analog risk task) significantly predicted greater risk of relapse to alcohol use when a drift diffusion model was applied to task behavior (as compared to traditional summary statistic estimates of task behavior) (Yuan et al. 2024). Using the same task during PET, a computational model also revealed slower behavioral updating as associated with lower striatal dopamine D2 receptor availability in individuals with methamphetamine use disorder, thus identifying a neural mechanism of this core behavioral deficit (Guttman et al. 2023).

While such theory-driven computational models are promising for uncovering specific addiction-related neuropsychological processes, an important further step will be to validate them as generalizable clinical biomarkers within the cross-validated machine learning framework (Yip et al. 2020). Naturalistic datasets should also facilitate the move to reliable clinical applications but may demand more of a data-driven approach that leverages larger datasets and models (e.g., LLMs) to handle complexity and identify predictive patterns (Agurto et al. 2025). Ultimately the development of clinical biomarkers is likely to involve a combination of these approaches, which can inform each other. By maintaining focus on cross-validated prediction of clinical variables in large datasets, the field can identify reliable markers with enhanced ecological/clinical (predictive) validity in an interactive feedback process.

Downloaded from www.annualreviews.org. Guest (guest) IP: 100.35.127.123 On: Mon, 13 Apr 2026 18:20:00

Supplemental References

Abi-Dargham A, Moeller SJ, Ali F, DeLorenzo C, Domschke K, et al. 2023. Candidate biomarkers in psychiatric disorders: state of the field. *World Psychiatry*. 22(2):236–62

Agurto C, Cecchi GA, King S, Eyigoz EK, Parvaz MA, et al. 2025. Speak and You Shall Predict: Evidence That Speech at Initial Cocaine Abstinence Is a Biomarker of Long-Term Drug Use Behavior. *Biological Psychiatry*

Bickel WK, Miller ML, Yi R, Kowal BP, Lindquist DM, Pitcock JA. 2007. Behavioral and neuroeconomics of drug addiction: Competing neural systems and temporal discounting processes. *Drug and Alcohol Dependence*. 90:S85–91

Curtis B, Giorgi S, Ungar L, Vu H, Yaden D, et al. 2023. AI-based analysis of social media language predicts

addiction treatment dropout at 90 days. *Neuropsychopharmacol.* 48(11):1579–85

Daw ND, O'Doherty JP, Dayan P, Seymour B, Dolan RJ. 2006. Cortical substrates for exploratory decisions in humans. *Nature*. 441(7095):876–79

Eickhoff SB, Milham M, Vanderwal T. 2020. Towards clinical applications of movie fMRI. *NeuroImage*. 217:116860

Goldstein RZ, Tomasi D, Rajaram S, Cottone LA, Zhang L, et al. 2007. Role of the anterior cingulate and medial orbitofrontal cortex in processing drug cues in cocaine addiction. *Neuroscience*. 144(4):1153–59

Goldstein RZ, Woicik PA, Moeller SJ, Telang F, Jayne M, et al. 2010. Liking and wanting of drug and non-drug rewards in active cocaine users: the STRAP-R questionnaire. *J. Psychopharmacol. (Oxford)*. 24(2):257–66

Grimm JW, Hope BT, Wise RA, Shaham Y. 2001. Incubation of cocaine craving after withdrawal. *Nature*. 412(6843):141–42

Gueguen MC, Schweitzer EM, Konova AB. 2021. Computational theory-driven studies of reinforcement learning and decision-making in addiction: what have we learned? *Current Opinion in Behavioral Sciences*. 38:40–48

Gueguen MCM, Anlló H, Bonagura D, Kong J, Hafezi S, et al. 2024. Recent Opioid Use Impedes Range Adaptation in Reinforcement Learning in Human Addiction. *Biol Psychiatry*. 95(10):974–84

Guttman Z, Mandelkern M, Ghahremani DG, Kohno M, Dean AC, London ED. 2023. Decomposing risky decision-making in methamphetamine use disorder: Behavioral updating and D2 dopamine receptors. *Drug Alcohol Depend*. 246:109860

Hasson U. 2025. Uncovering a Timescale Hierarchy by Studying the Brain in a Natural Context. *J. Neurosci.* 45(12):

Hasson U, Yang E, Vallines I, Heeger DJ, Rubin N. 2008. A Hierarchy of Temporal Receptive Windows in Human Cortex. *J. Neurosci.* 28(10):2539–50

Hser Y-I, Saxon AJ, Huang D, Hasson A, Thomas C, et al. 2014. Treatment retention among patients randomized to buprenorphine/naloxone compared to methadone in a multi-site trial. *Addiction*.

Downloaded from www.annualreviews.org. Guest (guest) IP: 100.35.127.123 On: Mon, 13 Apr 2026 18:20:00

109(1):79–87

Huang Y, Ceceli AO, Kronberg G, King S, Malaker P, et al. 2024. Association of Cortico-Striatal Engagement During Cue Reactivity, Reappraisal, and Savoring of Drug and Non-Drug Stimuli With Craving in Heroin Addiction. *AJP*. 181(2):153–65

Koban L, Wager TD, Kober H. 2023. A neuromarker for drug and food craving distinguishes drug users from non-users. *Nat Neurosci*. 26(2):316–25

Konova AB, Ceceli AO, Horga G, Moeller SJ, Alia-Klein N, Goldstein RZ. 2023. Reduced neural encoding of utility prediction errors in cocaine addiction. *Neuron*. 111(24):4058-4070.e6

Konova AB, Lopez-Guzman S, Urmanche A, Ross S, Louie K, et al. 2019. Computational Markers of Risky Decision-making for Identification of Temporal Windows of Vulnerability to Opioid Use in a Real-world Clinical Setting. *JAMA Psychiatry*

Kornfield R, Toma ,Catalina L., Shah ,Dhavan V., Moon ,Tae Joon, and Gustafson DH. 2018. What Do You Say Before You Relapse? How Language Use in a Peer-to-peer Online Discussion Forum Predicts Risky Drinking among Those in Recovery. *Health Communication*. 33(9):1184–93

Kronberg G, Ceceli AO, Huang Y, Gaudreault P-O, King SG, et al. 2025. Shared orbitofrontal dynamics to a drug-themed movie track craving and recovery in heroin addiction. *Brain*. 148(5):1778–88

Liu T, Giorgi ,Salvatore, Yadeta ,Kenna, Schwartz ,H. Andrew, Ungar ,Lyle H., and Curtis B. 2022. Linguistic predictors from Facebook postings of substance use disorder treatment retention versus discontinuation. *The American Journal of Drug and Alcohol Abuse*. 48(5):573–85

Parvaz MA, Moeller SJ, Goldstein RZ. 2016. Incubation of Cue-Induced Craving in Adults Addicted to Cocaine Measured by Electroencephalography. *JAMA Psychiatry*. 73(11):1127–34

Reimers N, Gurevych I. 2019. Sentence-BERT: Sentence Embeddings using Siamese BERT-Networks. *Proceedings of the 2019 Conference on Empirical Methods in Natural Language Processing and the 9th International Joint Conference on Natural Language Processing (EMNLP-IJCNLP)*, pp. 3982–92. Hong Kong, China: Association for Computational Linguistics

Shiffman S. 2009a. Ecological momentary assessment (EMA) in studies of substance use. *Psychol Assess*.

Downloaded from www.annualreviews.org. Guest (guest) IP: 100.35.127.123 On: Mon, 13 Apr 2026 18:20:00

21(4):486–97

Shiffman S. 2009b. How many cigarettes did you smoke? Assessing cigarette consumption by global report, Time-Line Follow-Back, and ecological momentary assessment. *Health Psychol*. 28(5):519–26

Sonkusare S, Breakspear M, Guo C. 2019. Naturalistic Stimuli in Neuroscience: Critically Acclaimed. *Trends in Cognitive Sciences*. 23(8):699–714

Taylor VA, Smith R, Brewer JA. 2022. App-Based Mindfulness Training Predicts Reductions in Smoking Behavior by Engaging Reinforcement Learning Mechanisms: A Preliminary Naturalistic Single-Arm Study. *Sensors (Basel)*. 22(14):

Team G, Anil R, Borgeaud S, Alayrac J-B, Yu J, et al. 2024. Gemini: A Family of Highly Capable Multimodal Models

Valdez D, Patterson MS. 2022. Computational analyses identify addiction help-seeking behaviors on the social networking website Reddit: Insights into online social interactions and addiction support communities. *PLOS Digital Health*. 1(11):e0000143

Yang G, King SG, Lin H-M, Goldstein RZ. 2023. Emotional Expression on Social Media Support Forums for Substance Cessation: Observational Study of Text-Based Reddit Posts. *Journal of Medical Internet Research*. 25(1):e45267

Yip SW, Kiluk B, Scheinost D. 2020. Toward Addiction Prediction: An Overview of Cross-Validated Predictive Modeling Findings and Considerations for Future Neuroimaging Research. *Biological Psychiatry: Cognitive Neuroscience and Neuroimaging*. 5(8):748–58

Yip SW, Scheinost D, Potenza MN, Carroll KM. 2019. Connectome-Based Prediction of Cocaine Abstinence. *AJP*. 176(2):156–64

Yuan W, Chen M, Wang D-W, Li Q-H, Yin Y-Y, et al. 2024. Computational markers of risky decision-making predict for relapse to alcohol. *Eur Arch Psychiatry Clin Neurosci*. 274(2):353–62

Downloaded from www.annualreviews.org. Guest (guest) IP: 100.35.127.123 On: Mon, 13 Apr 2026 18:20:00
